# Differential expression of neurogenes among breast cancer subtypes identifies high risk patients

**DOI:** 10.18632/oncotarget.6543

**Published:** 2015-12-10

**Authors:** Patricia Fernández-Nogueira, Paloma Bragado, Vanessa Almendro, Elisabet Ametller, Jose Rios, Sibgat Choudhury, Mario Mancino, Pedro Gascón

**Affiliations:** ^1^ Department of Medical Oncology, Hospital Clínic, Barcelona, Spain; ^2^ Institut d'Investigacions Biomediques August Pi i Sunyer Barcelona, Barcelona, Spain; ^3^ Department of Medicine, University of Barcelona, Barcelona, Spain; ^4^ Division of Medical Oncology, Department of Medicine, Harvard Medical School, Dana-Farber Cancer Institute, Brigham and Women's Hospital, Boston, MA, USA; ^5^ Medical Statistics Core Facility, IDIBAPS, (Hospital Clinic) Barcelona, Barcelona, Spain; ^6^ Biostatistics Unit, Faculty of Medicine, Universitat Autònoma de Barcelona, Barcelona, Spain

**Keywords:** breast cancer, neurogenes, neuropeptides, neurotransmitters, tumor microenvironment

## Abstract

The nervous system is now recognized to be a relevant component of the tumor microenvironment. Receptors for neuropeptides and neurotransmitters have been identified in breast cancer. However, very little is known about the role of neurogenes in regulating breast cancer progression. Our purpose was to identify neurogenes associated with breast cancer tumorigenesis with a potential to be used as biomarker and/or targets for treatment. We used three databases of human genes: GeneGo, GeneCards and Eugenes to generate a list of 1266 relevant neurogenes. Then we used bioinformatics tools to interrogate two published breast cancer databases SAGE and MicMa (*n*=96) and generated a list of 7 neurogenes that are differentially express among breast cancer subtypes. The clinical potential was further investigated using the GOBO database (*n*=1881). We identified 6 neurogenes that are differentially expressed among breast cancer subtypes and whose expression correlates with prognosis. Histamine receptor1 (*HRH1*), neuropilin2 (*NRP2*), ephrin-B1 (*EFNB1*), neural growth factor receptor (*NGFR*) and amyloid precursor protein (*APP*) were differentially overexpressed in basal and HER2-enriched tumor samples and syntaxin 1A (*STX1A*) was overexpressed in HER2-enriched and luminal B tumors. Analysis of *HRH1, NRP2*, and *STX1A* expression using the GOBO database showed that their expression significantly correlated with a shorter overall survival (*p* < 0.0001) and distant metastasis-free survival (*p* < 0.0001). In contrast, elevated co-expression of *NGFR, EFNB1* and *APP* was associated with longer overall (*p* < 0.0001) and metastasis-free survival (*p* < 0.0001). We propose that *HRH1, NRP2*, and *STX1A* can be used as prognostic biomarkers and therapeutic targets for basal and HER2-enriched breast cancer subtypes.

## INTRODUCTION

Intratumor heterogeneity refers to the coexistence of subpopulations of cancer cells diverging in their genetic, phenotypic, or behavioral characteristics within a given primary tumor, and between a given primary tumor and its metastases. Intratumor heterogeneity facilitates tumor progression and fosters the continuous adaptation and survival of the different tumor-propagating clones to the different microenvironments in which a tumor resides. A high degree of heterogeneity has been observed in many tumor types, including breast [1], prostate [2], ovarian [3], bladder [4], and pancreatic cancers [5, 6], as well as in glioma [7], chronic lymphocytic leukemia [8], multiple myeloma [9], and acute myeloid leukemia [5].

Insights from genomics have led to the identification of five molecular subtypes of breast cancer on the basis of gene expression patterns. Different molecular subtypes of breast cancer have different clinical outcomes and responses to chemotherapy [10]; therefore, intratumor heterogeneity represents a major challenge for the design of effective therapies. Intratumor heterogeneity results from the differentiation of stem-like cells and along with clonal selection, enables the propagation of the fittest clones for a given tumor microenvironment [11]. Breast cancer stem cells were initially identified using membrane antigenic markers. In 2003, Al-Hajj *et al.*[12] first described the existence of a CD44+CD24- subpopulation (hereinafter referred to as CD44+) in breast cancer with properties of tumor stem cells. The tumorigenic CD44+CD24−/low Lineage− population shares with normal stem cells the ability to proliferate extensively, and to give rise to diverse cell types with reduced developmental or proliferative potential. Moreover, this cell population is rich in cells capable of initiating tumors in immunosuppressed animals [12]. However, a large body of evidences has demonstrated that this phenotype is heterogeneous and not expressed in all breast cancers [13] [14]. In addition, Honeth *et al.* found CD44 expression predominantly on the cells surface membrane along with CD24 in the cytoplasm, and, most interesting, they showed that CD44 protein distribution or its degradation during tumor initiation and metastasis, may favor the enrichment of CD24 on the membrane [15]. Furthermore, It has been described that cancer cells can acquire a CD44+/CD24− phenotype through epithelial-to-mesenchymal transition (EMT) [16] Moreover, Meyer *et al.* hypothesized that an interconversion between the differentiated and stem-like phenotypes occurs in breast cancer and suggested that epithelial like CD44+/CD24+ can generate CD44+/CD24− cells during tumor initiation [17]. Therefore, the CD44+ is heterogeneous; nevertheless the expression of CD44 is correlated with a more aggressive phenotype in breast cancer and with poor outcome of patients with basal-like breast cancer [15].

Increasing evidence suggests that the nervous system itself, as well as neurotransmitters and neuropeptides present in the tumor microenvironment, play a role in orchestrating tumor progression. Theoretically, just as tumors induce the formation of new blood vessels (angiogenesis) [18] and lymph vessels (lymphangiogenesis) [19] by secreting different factors, tumors may also induce the formation of new nerve endings, (neoneurogenesis) [20] by secreting neurotrophic factors and axonal guidance molecules. In this scenario, the neuroendocrine system would play a key role in cancer progression and metastasis. In fact, colon tumors that express neuroendocrine markers have poor prognosis [21]. Synaptophysin, a protein found in neuroendocrine cells and in virtually all neurons in the central nervous system participating in synaptic transmission, has been detected in breast [22], colon [23], prostate [24], and brain [25] tumors as well as melanoma [26], supporting the idea that nerve fibers infiltrate tumors. Furthermore, the release of neurotrophic factors such as norepinephrine, dopamine, and substance P appears to stimulate the growth of nerve fibers inside tumors [27–29]. Nerve endings in turn release factors that stimulate the migratory activity of tumor cells and promote metastasis [20]. In addition, netrin-1 (an axonal guidance molecule) and its receptor neogenin are involved in maintaining adhesion between basal and luminal cells in adjacent cap cells of the mammary gland terminal end buds [30]. Netrin-1 regulates invasion and migration of breast epithelial tumor cells [31] and promotes the survival of tumor cells in metastatic breast cancer [32]. In this context, we recently demonstrated that netrin-1 negatively regulates the expression of stem cell markers (Nanog, Oct3/4, and CRIPTO-1) in human embryonic carcinoma cells and mouse embryonic stem cells [33]. Furthermore, individual studies have linked various aspects of cancer biology to certain neurotransmitter receptors, such as the beta-2 adrenergic receptor [34] and the tachykinin NK1 receptor [35] as well as to soluble factors such as bradykinin [36]. Substance P, an inflammatory neuropeptide and its receptor NK1, are overexpressed in breast cancer [37]. Our group found that blocking substance P signaling promotes death in breast cancer cells [38]. Moreover, we have shown that substance P promotes cancer progression and drug resistance by contributing to persistent HER2 activation [35]. However, the role of neurotransmitters and their receptors in breast cancer progression is still unclear. It could be hypothesized that, analogous to the proinflammatory cytokines, certain neurotransmitters and neuropeptides in the microenvironment may promote tumor progression by selecting certain specifically responsive clones. The nervous system could exert direct and indirect control of tumor progression mainly through modulation of the immune system [39].

To characterize the role of neurotransmitters, neuropeptides, neurotrophic factors, and axonal guidance molecules in breast cancer progression, we analyzed the expression of several neurogenes in breast cancer patients and in CD44 and CD24 expression databases. Using bioinformatics tools, we identified 7 neurogenes that are differentially expressed in different breast cancer subtypes. The expression of 6 of these neurogenes correlates with prognosis, so we propose that they can be used as potential targets for novel therapeutic approaches against signaling pathways activated in breast cancer stem cells.

## RESULTS

### Identification of neurotransmitters, neuropeptides, axonal guidance molecules, neurotrophic factors, and/or their receptors differentially expressed in CD44+ and CD44-CD24+ breast cancer cells

**Figure 1:**
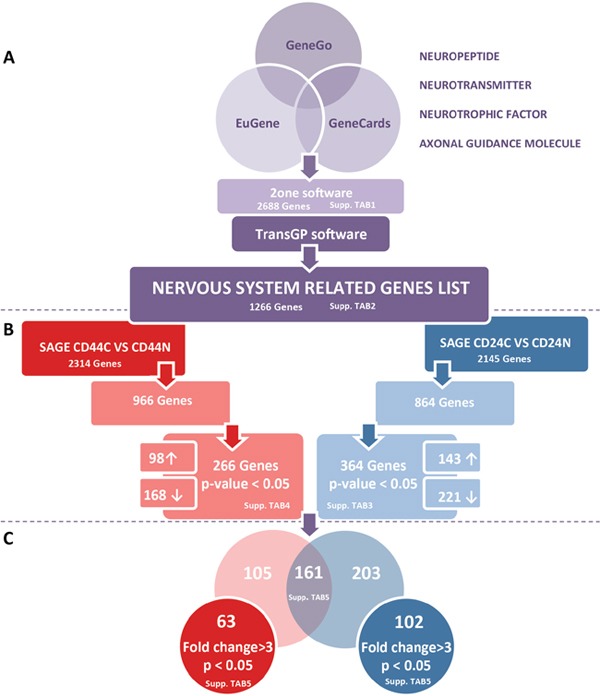
A schematic overview of the approach used in this study To focus on molecules related to neuronal processes, we compiled a list of genes related to the function of the nervous system. We searched for the terms “axon guidance”, “neuropeptide”, “neurotransmitter”, “neurotrophic factor”, and “neurotrophic molecule” in different databases (GeneCard, Eugene, GeneGo). Genes categorized under these keywords were collected and filtered with 2ONE to delete duplicates. Given the categorical redundancy for some genes, we next confirmed the function of each gene and its direct relationship with neuronal processes and the study context to obtain a final list comprising 1266 genes **A.** The SAGE-Seq profiles for CD44+ and CD24+ normal (CD44N and CD24N) and cancer cells (CD44C and CD24C) are publicly available. To investigate which neurogenes were differentially expressed in CD44+ and CD24+ cells within normal breast epithelium and cancer tissue, we filtered our neurogene list from the different SAGE-Seq profiles. By comparing the genes differentially expressed in CD24+ normal and tumor cells (CD24N vs CD24C), and those differentially expressed in CD44+ normal and tumor cells (CD44N vs CD44C), we obtained the genes differentially expressed only in CD44+ cancer cells and only in CD24+ cancer cells, resulting in a final list of 266 and 364 neurogenes differentially expressed in CD44+ and CD24 tumor cells, respectively (*p* < 0.05) **B.** From these cell-type specific gene lists, we analyzed how many were commonly represented in CD44+ and CD24+ tumor cells, and which ones were specific to different cell phenotypes. Genes were further filtered in expression (≥ 3-fold changes) to obtain our final lists of 63 neurogenes differentially expressed in CD44+ tumor cells and 102 neurogenes differentially expressed in CD24+ tumor cells **C.**

Using three searchable, integrated databases of human genes GeneGo (www.portal.genego.com), GeneCards (www.genecards.org), and Eugenes (www.eugenes.org) we generated a preliminary list of 1266 neurogenes, (Supplementary Table S2 and Figure 1A).

To identify the neurogenes differentially expressed between breast cancer CD44+ population and the more differentiated CD44-CD24+ (hereinafter referred to as CD24+) cells, we used the databases containing the Serial Analysis of Gene Expression (SAGE) expression libraries obtained in the laboratory of Dr. Kornelia Polyak (in collaboration with Dr. Kornelia Polyak, for more details on the procedure see Shipitsin, M. *et al* [40]). These libraries were created from different mammary epithelium cell populations obtained from normal tissue from patients who underwent reduction mammoplasty and tumor cell subpopulations obtained from breast cancer patients.

We correlated the SAGE database expression patterns with our list of neurogenes to identify neurogenes that are differentially expressed in breast cancer tissue compared with healthy epithelium and that are differentially expressed in each cell subtype (CD44+ vs. CD24+). These correlations gave us a more detailed view of the possible specific functions of these neurogenes in each subpopulation of breast cancer cells.

Using SAGE-Seq libraries, we identified 2,145 genes that are differentially expressed in CD24+ cancer cells compared to CD24+ normal breast epithelium cells. Of these, 864 genes also figured in our list of 1,266 neurogenes. The tool's default p-value cutoff of 0.05 combined with the false discovery rate to eliminate false positives, generated a list of 364 genes differentially expressed between cancerous and normal CD24+ cells (Supplementary Table S3); in 143 of these CD24 expression was higher in cancerous cells than in normal cells and in 221, CD24 expression was lower in cancerous cells than in normal cells (Figure 1B right).

Moreover, using SAGE-Seq libraries, we identified 2314 genes differentially expressed in CD44+ cancer cells compared to CD44+ normal breast epithelium cells. Of these, 966 also figured in our list of neurogenes. The tool's default p-value cutoff of 0.05 combined with the false discovery rate, generated a list of 266 genes differentially expressed between cancerous and normal CD44+ cells (Supplementary Table S4); in 98 CD44 expression was higher in cancerous cells than in normal cells and in 168 genes CD44 expression was lower in cancerous cells than in normal cells (Figure 1B left).

Comparing the lists of neurogenes differentially and significantly expressed in CD24+ cancer cells to CD24+ normal breast epithelium cells (364 genes) and in CD44+ cancer cells to CD44+ normal breast epithelium cells (266 genes), we identified 161 genes differentially expressed in both cell subtypes, 203 genes differentially expressed only in CD24+, and 105 genes differentially expressed only in CD44+ (Supplementary Table S5 and Figure 1C).

To identify genes that varied widely, we set an arbitrary fold change cutoff of >3. This way, we found 102 genes differentially expressed in CD24+ cancer cells compared to CD24+ normal breast epithelium cells, and 63 genes differentially expressed in CD44+ cancer cells compared to CD44+ normal breast epithelium cells (Supplementary Table S5 and Figure 1C).

**Figure 2:**
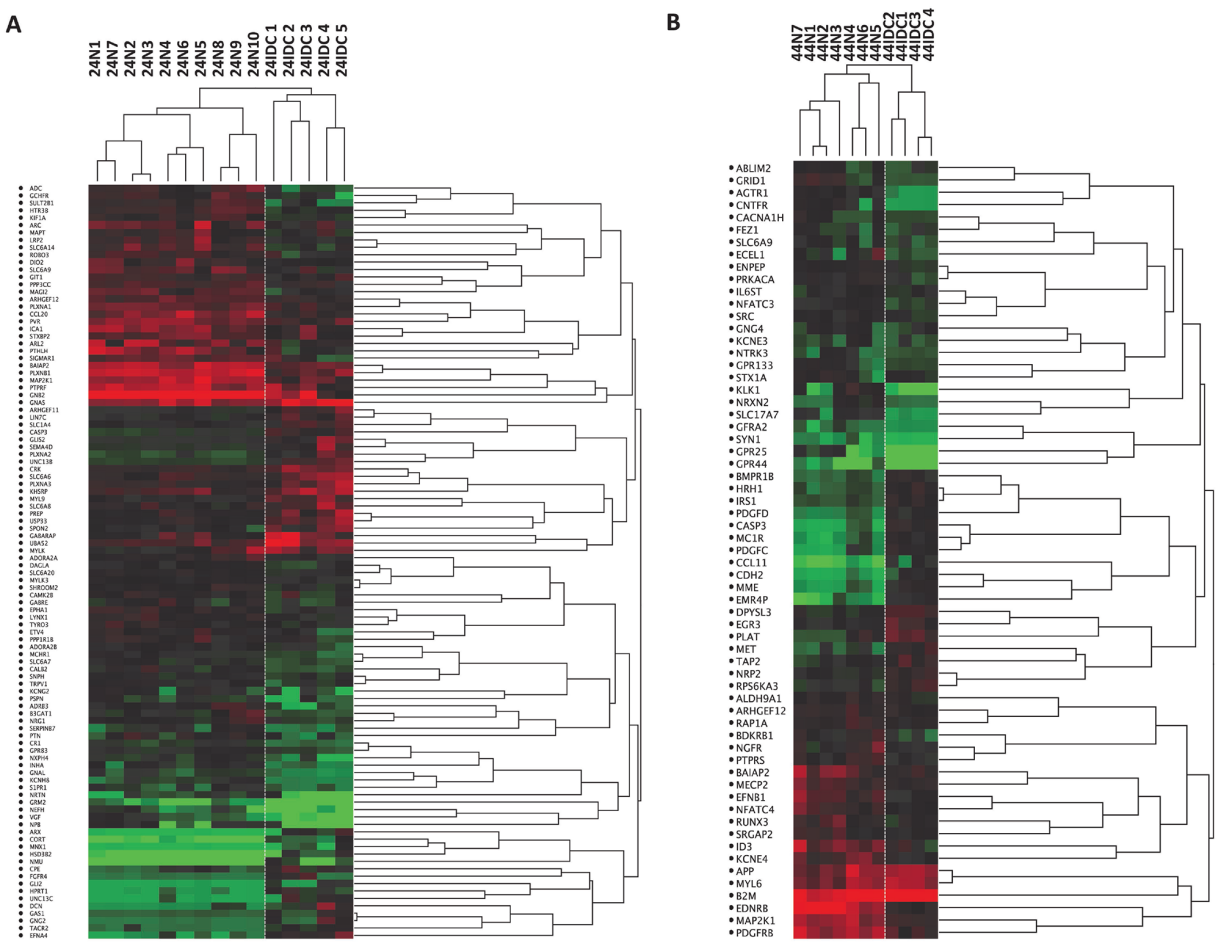
Cell-type specific differences in gene expression Dendrogram depicting relatedness of SAGE libraries prepared from CD24+ **A.** and CD44+ **B.** cells. Hierarchical clustering was applied to SAGE data for the indicated libraries and clustering heat maps are shown here. Each row represents a tag and is labeled with the symbol of the gene that best matches that tag (or “no match” if no matching transcripts was found) Red and green indicate high and low expression levels, respectively. N, Normal; IDC, Invasive Ductal Carcinoma.

Figure 2 shows the differential expression of the selected genes represented with a supervised hierarchical clustering (based closely on the average-linkage method of Sokal and Michener) (Figure 2).

### Gene set expression in human breast cancer subtypes

Previous studies have identified CD44+ cells role in enhancing breast cancer cell migration and invasion [41]. CD44+ cells are associated with the most clinically aggressive breast cancer subtypes (triple-negative and HER2-enriched) [42, 43]. Moreover, CD44+ cells have a mesenchymal phenotype and express enzymes for drug detoxification that confer resistance to chemotherapy [42],[44, 45]. For these reasons, we focused on the analysis of the neurogenes differentially expressed in CD44+ cells and decided to leave the genes differentially expressed in CD24+ cells for future studies.

**Figure 3:**
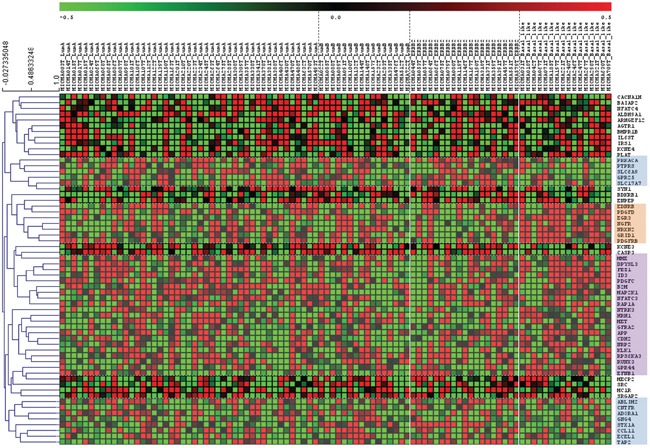
Neurogenes expression among breast cancer subtypes (MicMa Database) Tumor gene expression levels for 96 patients with different breast cancer subtypes Rows represent microarray probes corresponding to the selected genes (neurogenes differentially expressed in CD44+ cells) and columns represent patients. 3 independent gene set were identified and gathered. Orange square: genes upregulated in basal like patients: *EDNRB, PDGFD, EGR3, NGFR, NRXN2, GRID1, PDGFRB*. Blue squares: genes downregulated in basal like patients and upregulated in HER-2 enriched patients: *PRKACA, PTPRES, SLC6A9, GPR25, SLC17A7, ABLIM2, CNTFR, ADORA1, GNG4, STX1A, CCL11, ECEL1, TAP2*. Violet square: genes upregulated in basal and HER2-enriched patients: *MME, DPYSL3, FEZ1, ID3, PDGFC, B2M, MAP2K1, NFATC3, RAP1A, NTRK3, HRH1, MET, GFRA2 APP, CDH2, NRP2, KLK1, RPS6KA3, RUNX3, GPR44, EFNB1*. Red and green indicate high and low expression levels, respectively.

We correlated the expression of the 63 genes differentially expressed between CD44+ cancer cells and CD44+ normal tissue with clinical and pathological data, using the information from the MicMa breast cancer patients cohort database, which includes data on molecular expression and clinical information on 96 breast cancer patients followed up for 10 years (http://www.ncbi.nlm.nih.gov/geo, accession number GSE19425) (Figure 3). Focusing on the expression of this genes in basal and HER2-enriched subtypes we found 3 sets of genes, one where genes were upregulated in basal-like patients, one in which genes were upregulated in HER2-enriched patients and a third set of genes that were upregulated in basal and HER2-enriched patients (Figure 3).

**Figure 4:**
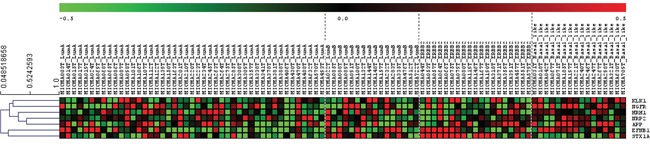
Expression among breast cancer subtypes of our selected neurogenes (MicMa Database) Selected neurogenes (*KLK1, NRP2, EFNB1, STX1A, NGFR, HRH1, APP*) expression levels for 96 patients with different breast cancer subtypes. Rows represent microarray probes corresponding to the selected genes and columns represent patients. Red and green indicate high and low expression levels, respectively.

We used then an independent database “Gene expression-Based Outcome for Breast Cancer” (GOBO) [46], which includes a cohort of 1881 breast cancer patients to validate the data obtained in the MicMa analysis. After these two analyses we obtained a final list of 7 neurogenes that are differentially expressed among breast cancer subtypes (Figure 4, Table 1).

**Table 1:** List of 7 specific neurogenes differentially expressed in CD44+ cells and breast cancer subtypes

Gene	Aliases & Descriptions	UniGene Cluster	Entrez Gene Cyto Band
*KLK1*	Kallikrein 1	Hs.123107	chr19q13.33
*NGFR*	Nerve Growth Factor Receptor	Hs.415768	chr17q21-q22
*HRH1*	Histamine Receptor H1	Hs.1570	chr3p25
*NRP2*	Neuropilin 2	Hs.471200	chr2q33.3
*APP*	Amyloid Beta (A4) Precursor Protein	Hs.434980	chr21q21.2|21q21.3
*EFNB-1*	Ephrin-B1	Hs.144700	chrXq13.1
*STX1A*	Syntaxin 1A	Hs.647024	chr7q11.23

6 of the 7 selected neurogenes were upregulated in basal-related tumors (the closely associated basal-like and/or HER2-enriched breast cancer phenotypes [47]) compared to luminal A and luminal B breast tumor subtypes (Figure 4). Kallikrein 1(*KLK1*), histamine receptor 1 (*HRH1*), neuropilin 2 (*NRP2*), amyloid precursor protein (*APP*) and ephrin- B1 (*EFNB1*) were clearly differentially overexpressed in basal and HER2-enriched tumor samples, neural growth factor receptor (*NGFR*) expression in the MicMa cohort was associated to basal-like subtypes (Figure 4). On the other hand, syntaxin 1A (*STX1A*) was overexpressed in luminal B and HER2-enriched patients (Figure 4).

To validate the association of these neurogenes that we had seen were differentially expressed among breast cancer subtypes in the MicMa cohort with clinical outcome, we used again the online database GOBO [46]

**Figure 5:**
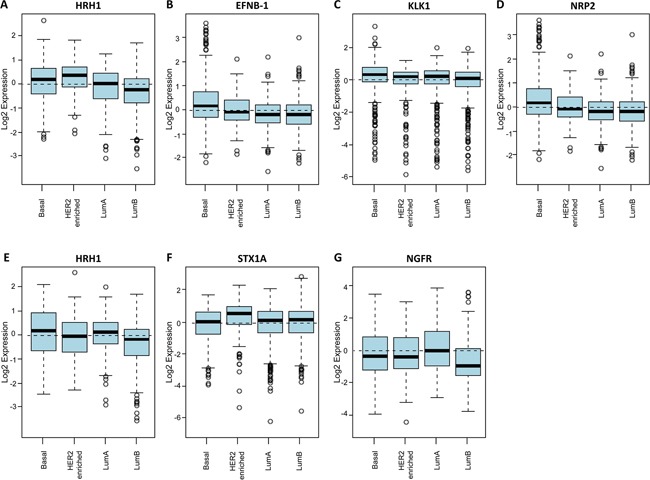
Neurogenes expression related to breast cancer molecular subtypes using the GOBO database Box plot of *HRH1* **A.** *EFNB1* **B.** *KLK1* **C.** and *NRP2* **D.** gene expression for tumor samples stratified according to HU subtypes (all tumors untreated, *p* = < 0.00001). Box plot of *APP* **E.** *STX1A* **F.** and *NGFR* **G.** gene expression for tumor samples stratified according to PAM50 subtypes (all tumors untreated, *p* = < 0.00001) (HU subtypes referred to Hu 306 gene set [76] and PAM50 referred to PAM 50 gene set are two independent intrinsic gene signatures retrieved for molecular subtype prediction [77]).

Applying the Gene Set Analysis (GSA) we found that elevated expression of *HRH1, EFNB1, KLK1, NRP2*, and *APP* was associated with the basal-like subtypes of breast cancer (Figure 5A–5E) as predicted by the MicMa cohort. In addition, *HRH1* was also upregulated as expected in HER2-enriched tumors (Figure 5A). Gene set analysis for *STX1A* expression also corroborated the results collected using the MicMa database: high *STX1A* expression associates with HER2-enriched and luminal B breast cancer subtypes (Figure 5F). However, *NGFR* expression on the GOBO was upregulated in luminal A patients and not in basal-like (Figure 5G). Therefore, it didn't correlate with the MicMa data. Nevertheless, in the case of *NGFR* it had been previously shown that it can be used as a marker for basal-like breast carcinomas associated with good prognosis [48], which was in agreement with our results in the MicMa cohort.

**Figure 6:**
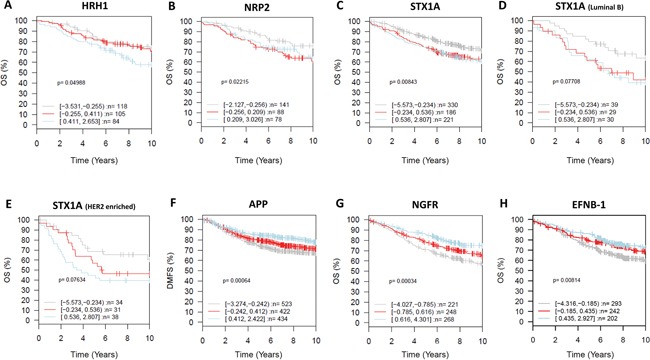
Clinical relevance of the neurogenes differentially expressed among breast cancer subtypes (GOBO database) GSA for selected neurogenes, prognostic value (X-axis representing time in years). Kaplan-Meier analysis using overall survival (OS) (HRH1- all tumors untreated **A.** NRP2 - all tumors untreated **B.** STX1A all tumors untreated **C.** STX1A Luminal B **D.** STX1A Her2-enriched **E.** NGFR - all tumors untreated **G.** EFNB1 all tumors untreated **H.**) and distant metastasis free survival (DMFS) (APP – all tumors untreated **F.**) as endpoint and 10-year censoring as displayed in GOBO.

We then analyzed the correlation of the expression of these neurogenes with survival. Overexpression of *HRH1, NRP2*, and *STX1A* correlated with shorter overall survival and can therefore be considered indicators of poor prognosis (Figure 6A, 6B and 6C). High *STX1A* expression in luminal B and HER2-enriched tumors correlated with even lower overall survival (Figure 6D–6E) and can therefore be considered an indicator of poor prognosis for this breast cancer subtypes. By contrast, *APP, NGFR* and *EFNB1* expression was associated with longer metastasis-free periods and higher overall survival and can therefore be considered indicators of good prognosis (Figure 6F–6H). Analysis of *KLK1* expression in the GOBO database showed that its expression didn't correlate with survival so it cannot be used to stratify patients.

**Figure 7:**
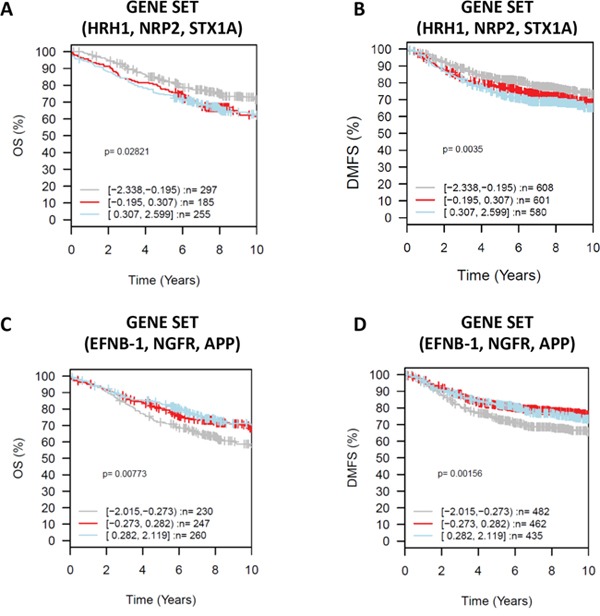
Gene Set Analysis (GSA) for selected neurogenes in gene sets, prognostic value Kaplan-Meier analysis using overall survival (OS) (*HRH1, NRP2, STXA1* gene set analysis **A.** *EFNB1, NGFR, APP* gene set analysis **C.**) and distant metastasis free survival (DMFS) (*HRH1, NRP2, STXA1* gene set analysis **B.** *EFNB1, NGFR, APP* gene set analysis **D.**) as endpoint and 10-year censoring as displayed in GOBO. X axis representing time in years.

High expression of *HRH1, NRP2*, and *STX1A* together as a gene set, correlated with worse outcome in untreated tumors and with shorter metastasis-free periods (Figure 7A–7B). Although the expression of each of these three genes was independently associated with basal-related cancer and correlated with shorter overall survival (Figure 5 and 6), analyzing them together as a set is more powerful in predicting clinical outcome. Elevated co-expression of *EFNB1, NGFR* and *APP* in breast cancer tumors was associated with longer overall, metastasis-free, and relapse-free survival (Figure 7C–7D).

These findings support an important role for neurogenes in breast cancer progression. We propose that this set of 6 neurogenes *HRH1, NRP2, STX1A, APP, EFNB1* and *NGFR* can be used to stratify patients according to their predicted clinical outcome. In addition, *HRH1* and *NRP2* should be considered interesting targets for the treatment of basal-related breast cancer subtypes, and *STX1A* could be an interesting target for the treatment of the luminal B and HER2-enriched subtypes.

## DISCUSSION

Intratumor phenotypic and functional heterogeneity arise among cancer cells as a consequence of genetic alterations, reversible changes in cell properties, and microenvironmental diversity. It is becoming progressively more evident that interactions between carcinoma cells and the tumor microenvironment are an essential part of tumor biology [49]. In particular, it has recently been reported that the host neuroendocrine system can affect the activity of cells present in it [50]. However, little is known about the influence of the nervous system on cancer progression. In response to psychological and/or social pressures, the nervous system releases factors that might affect both cancer progression and the efficiency of drugs to treat cancer. For these reasons, it might be interesting to re-examine the methods for testing drugs *in vivo*, which currently involve efforts to ensure that animals are kept in stress-free environments.

Similarly, conventional cancer treatments such as chemotherapy or even surgical ablation commonly do not take into account the complexities added by nervous system and life-style factors that can affect the tumor host microenvironment. An imbalance between stimulatory and inhibitory nervous system factors might influence the initiation, progression, and especially the relapse of the most common human cancers [51]. For these reasons, we focused on identifying neurogenes that might be involved in breast cancer progression.

Through several independent analyses of the data, we identified a set of 6 genes that can be used as prognostic markers for different breast cancer subtypes. The robustness of our methods makes us confident that these genes are important in breast cancer progression.

The neurogenes *APP* and *EFNB1* are upregulated in basal and HER2-enriched breast tumors meanwhile *NGFR* is upregulated only in basal-like breast tumors. In addition, *EFNB1* and *NGFR* were downregulated in CD44+ cancer cells versus normal cells. Furthermore, *APP, NGFR and EFNB1* expression correlated with better overall survival. APP is a single transmembrane protein that has been linked to Alzheimer disease [52]. Recently, it has been reported that several types of cancers have increased expression of APP, which correlated with increase cancer cell proliferation [53–57]. In breast cancer, it has been recently shown that APP promotes cell proliferation and favors breast cancer cells motility. Furthermore, APP expression was recently positively associated with androgen receptor (AR) expression, Ki-67 and increased risk of recurrence in oestrogen receptor (ER)-positive patients [57]. In our study, APP expression correlates with better overall survival in ER negative patients and we propose that its expression identifies a subgroup of basal-like patients with good prognosis. EFNB1 is a type I membrane protein and a ligand of Eph-related receptor tyrosine kinases [58]. It has been described to play a role in cell adhesion and in the development or maintenance of the nervous system [59]. In breast cancer, in agreement with our data, it was recently reported that high expression of EFNB1 was positively correlated with lymph node metastasis and with the presence of HER2 receptor. However, in this study, it was also demonstrated that enhanced EFNB1 expression was associated with shorter overall survival [60]. On the contrary, in our study, high expression of EFNB1 correlated with better overall survival and longer metastasis free periods. Therefore, we proposed, that elevated expression of EFNB1 in basal and HER2-enriched patients identifies a good prognosis subpopulation. NGFR is a transmembrane protein receptor for the neurotrophin family. It has been previously reported that it can act as tumour suppressor in different types of cancer. In breast cancer studies using breast cancer cell lines had shown that NGFR signalling regulates breast cancer cells survival. Furthermore, NGFR expression has been previously associated with basal like and luminal B tumors [48]. Furthermore, in another study, Reis-Filho *et al.* suggested that NGFR identified a subgroup of basal-like breast cancers with good prognosis [61]. Our results are in agreement with these data and therefore, we propose that NGFR can be used as a good prognosis indicator for basal patients.

The neurogenes *HRH1* and *NRP2* were upregulated in basal-related breast tumors and CD44+ cancer cells and another, *STX1A*, was upregulated in luminal B and HER2-enriched tumors. Although other authors have already reported that *HRH1* and *NRP2* were involved in breast cancer development and progression [62, 63], to our knowledge, the overexpression of these genes had not been correlated with basal-related breast cancer subtypes. On the other hand *STX1A* had not been related to breast cancer. The overexpression of this gene in the HER2-enriched and luminal B subtypes and its correlation with worse prognosis suggest it might be a promising target for these breast cancer subtypes.

Histamine is a ubiquitous messenger molecule released by mast cells, enterochromaffin-like cells, and neurons. Its various actions are mediated by the histamine receptors HRH1, HRH2, HRH3, and HRH4. Histamine and its HRH1 receptor are involved in breast tumor development and metastasis [64]. In gastric cancer, HRH1 is expressed in circulating tumor cells [65] and can be used as a biomarker to predict which patients have minimal residual disease and therefore a higher risk of developing metastases. Moreover, in melanoma, HRH1 inhibition delays tumor growth and prevents lung metastasis [66]. Our data also suggest that HRH1 might be important for cancer progression. We hypothesize that basal-like tumors with high expression of HRH1 will be more aggressive and result in poorer outcomes and that HRH1 inhibitors might be a promising therapy for these tumors.

The *NRP2* gene encodes a member of the neuropilin family of receptor proteins, NRP2. This protein is a receptor for the proteins semaphorin-3C (SEMA3C) and semaphorin-3F (SEMA3F) and also plays a role in regulating angiogenesis, principally by interacting with vascular endothelial growth factor (VEGF) [67]. In addition to VEGF, NRP2 can bind many other growth factors such as transforming growth factor-beta (TGF-β), which may contribute to angiogenesis as well as to cancer cell survival and proliferation [68]. NRP2 is overexpressed in many cancer cell types, including astrocytoma, neuroblastoma, melanoma, and pituitary and ovarian cancers. Furthermore, NRP2 plays a role in breast cancer metastasis by promoting migration and invasion [69]. Therefore, we hypothesize that NRP2 expression might promote tumor angiogenesis and metastasis in basal-like breast cancer.

Finally, STX1A is a member of the syntaxin superfamily [70]. Syntaxins are nervous system-specific proteins implicated in the docking of synaptic vesicles with the presynaptic plasma membrane. STX1A expression has been correlated with Williams's syndrome, cystic fibrosis [71] and Alzheimer's disease [72]. Very few information is available about the role of STX1A in cancer in general or in breast cancer in particular, although it forms part of the SNARE complexes, which seem to play a role in cancer cell migration [73]. Furthermore, it was recently described that STX1A inhibition promotes glioblastoma tumor growth [74]. Moreover, in neurons, STX1A interact with netrin-1 receptors to promote chemoattraction in migrating neurons. Thus, we proposed that STX1A expression in HER2-enriched and luminal B breast cancer might favor the migration of cancer cells and invasion of the surrounding tissue.

Our analysis of *HRH1, NRP2*, and *STX1A* expression using the GOBO database corroborated the overexpression of *HRH1* and *NRP2* in basal-like and HER2-enriched cancer subtypes, while *STX1A* was overexpressed only in HER2-enriched and luminal B subtypes. Moreover, the expression of these genes also significantly correlated with a shorter overall survival (OS) (*p* < 0.0001) and distant metastasis-free survival (*p* < 0.0001) in the Kaplan-Meier analysis. Therefore, therapies targeting *HRH1, STX1A*, and *NRP2* might improve outcomes in basal-related breast cancer.

In conclusion, we have identified a set of neurogenes whose expression correlate with different breast cancer subtypes that are promising candidates as biomarkers to classify patient's outcome and might be promising targets for novel treatment approaches.

## MATERIALS AND METHODS

### Human genes databases and neurogene list generation

To generate a preliminary list of neurogenes, we used three searchable, integrated databases of human genes GeneGo (www.portal.genego.com), GeneCards (www.genecards.org), and Eugenes (www.eugenes.org) provided a common summary of gene and genomic information from eukaryotic organism databases. We used the search terms “axonal guidance molecule”, “neuropeptide”, “neurotransmitter”, “neurotrophic factor”, and “neurotrophic molecule” to consult those databases.

After using “2one” software tool (http://bioinformatics.fcrb.es/anntools/toone.php) to detect and delete genes repeated in the results of the three searches, we had a final list of 2688 genes.

We used TransGP software (http://bioinformatics.idibaps.org/anntools/transgp.php) to integrate and manage the omics information of the genes in our final list, including Refseq, Protein ID, Transc_Refseq, Unigene Cluster, Entre Gene Cyto Band, and Aliases & Descriptions (Supplementary Table S1). Using this information and considering the biological relevance of each gene in our project, we reduced the number of genes to be included in further screening and analysis to 1266 (Supplementary Table S2 and Figure 1A).

### Gene expression data and patient samples

To identify the neurogenes differentially expressed between less differentiated breast cancer cells (CD44+) and more differentiated cells (CD24+), we took advantage of the databases containing the Serial Analysis of Gene Expression (SAGE) expression libraries obtained in the laboratory of Dr. Kornelia Polyak (in collaboration with Dr. Kornelia Polyak, for more details on the procedure see Shipitsin, M. *et al* [40].

Ninety-six patients were included in this study. Fresh-frozen tumor biopsies were collected from patients included in the “Oslo Micrometastasis Project” from 1995 to 1998. A summary of the MicMa cohort with clinical and pathological data was published and is available in the original papers Hege G. Russnes, *et al.* Sci Transl Med 2010 [75].

### Prognostic validation

We used the Gene expression-based Outcome for Breast cancer Online (GOBO) tool for prognostic validation of individual genes and as well as of gene sets in a pooled breast cancer dataset comprising 1881 samples [46]. Association with outcome was investigated by Kaplan-Meier analysis using overall survival and distant metastasis-free survival as endpoints and 10-year censoring.

### Statistical analyses

To identify the neurogenes differentially expressed between breast cancer CD44+ population and the more differentiated CD24+ cells, we arbitrary set the significance level at p < 0.05. Moreover, False Discovery Rate (FDR) adjustment was applied to control and manage the data reducing the expected proportion of false positives among all suitable genes. Statistical tests were conducted using Graphpad Prism 6 software.

## SUPPLEMENTARY TABLES












