# Supplementary material for: Differential expression of neurogenes among breast cancer subtypes identifies high risk patients
Source: Oncotarget. 2015 Dec 10;7(5):5313–26. doi: 10.18632/oncotarget.6543 (PMC4868688; doi:10.18632/oncotarget.6543)
Supplement: Supplementary file 1 [file oncotarget-07-5313-s001.pdf]

SUPPLEMENTARY TABLES

Supplementary Table S1: A list of 2688 neurogens has been obtained using 3 searchable, integrated databases of human genes (GeneGo (www.portal.genego.com), GeneCards (www.genecards.org), and Eugenes (www.eugenes.org)). The omics information of the genes, including Refseq, Protein ID, Transc_Refseq, Unigene Cluster, Entre Gene Cyto Band, and Aliases & Descriptions, were integrated and managed by TransGP software (http://bioinformatics.idibaps.org/anntools/transgp.php)

Supplementary Table S2: A final list of 1266 neurogens obtained from DATABASE1 after filtering according to the biological relevance of each gene

Supplementary Table S3: A list of 364 neurogenes differentially expressed in CD24+ cells (221 neurogenes underexpressed; 143 neurogenes overexpressed)

Supplementary Table S4: A list of 266 neurogenes differentially expressed in CD44+ cells (168 neurogenes underexpressed; 98 neurogenes overexpressed)

Supplementary Table S5: Using an arbitrary fold change cutoff of > ± 3, 63 neurogenes are differentially expressed in CD44+ cancer cells compared to CD44+ normal breast epithelium cells (21 neurogenes >+3 in red; 42 neurogenes >-3 in green) and 102 neurogenes are differentially expressed in CD24+ cancer cells compared to CD24+ normal breast epithelium cells (36 neurogenes >+3 in red; 66 neurogenes >-3 in green). FC: Fold Change; FDR: False Discovery Rate
